# Concurrent Chemoradiation With 5-Fluorouracil and Mitomycin in Squamous Cell Carcinoma of the Rectum

**DOI:** 10.7759/cureus.17518

**Published:** 2021-08-28

**Authors:** Ashish K Sethi

**Affiliations:** 1 Medical Oncology, Allegheny Health Network, Pittsburgh, USA

**Keywords:** 5-fluorouracil, anal cancer, mitomycin, definitive chemoradiotherapy, human papillomavirus (hpv), rectal squamous cell carcinoma

## Abstract

Rectal carcinoma-squamous type is infrequently seen. Etiopathogenesis, prognosis, and therapeutic management of rectal squamous cell carcinoma (SCC) are not clearly defined. Rectal SCC is now approached with definitive upfront chemoradiotherapy (CRT), with 5-fluorouracil (5-FU) and mitomycin with a goal to avoid surgery. However, its management is planned based on histology features regardless of the localization of SCC rectal cancer. We present a case of a 47-year-old Caucasian female with rectal SCC who is under remission for two years after being treated with upfront chemoradiation with mitomycin and 5-fluorouracil (5-FU).

## Introduction

Rectal squamous cell carcinoma (SCC) is a very rare entity in colorectal cancers. The first rectal SCC was seen in the year 1933 [1]. The incidence of rectal SCC has been now reported to be around 0.10-0.25 per 1000 colorectal cancers [1]. There has been significant heterogeneity in the treatment regimens of rectal SCC, with the optimal management yet to be clarified. The standard protocol for SCC of the rectum is surgical treatment. Due to its aggressive nature and late presentation, surgery in this subtype has been always regarded as the best possible chance for remission. We present a case of a 47-year-old Caucasian female with rectal SCC who is under remission for two years after being treated with upfront chemoradiation with mitomycin and 5-fluorouracil (5-FU).

## Case presentation

**Figure 1:**
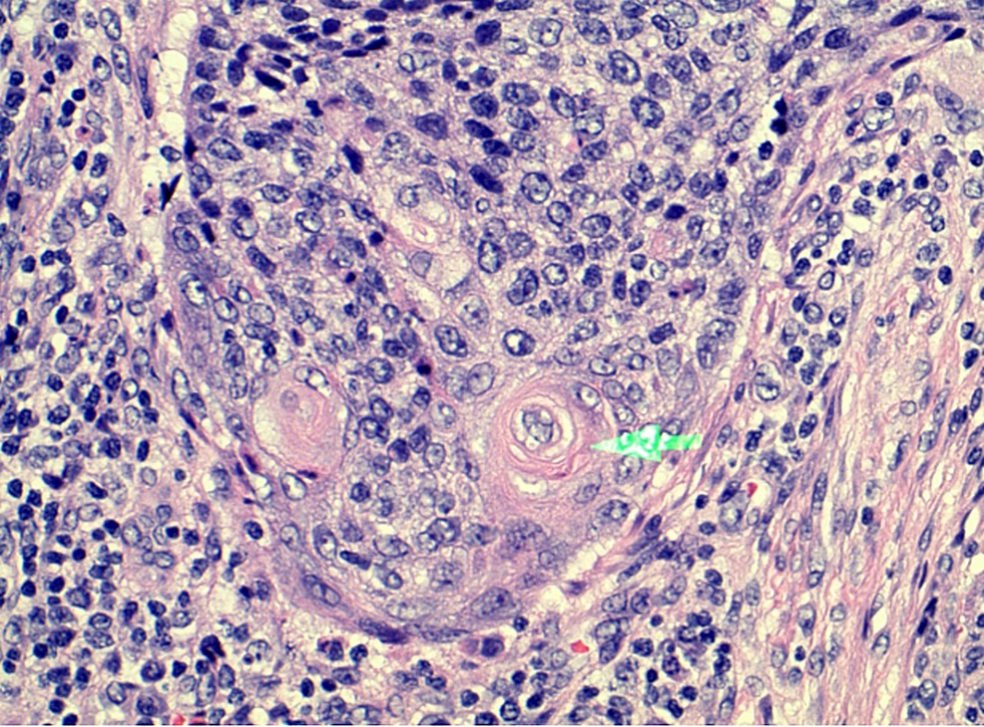
Histology and immunohistochemistry have verified the presence of a rectal SCC SCC: squamous cell carcinoma Makadia et al. granted permission to use this image [2].

A 47-year-old Caucasian female presented with complaints of mild diffuse abdomen pain, fatigue, and constipation over two months. She reported that her symptoms were associated with intermittent episodes of rectal bleeding. The patient was a nonsmoker and had no history of alcohol consumption. Her review of systems was negative for weight loss, night sweats, fever, nausea, vomiting, shortness of breath, and chest pain. Digital rectal examination (DRE) revealed a rectal mass. All laboratory tests were within normal range. Computed tomography (CT) of the abdomen and pelvis was positive for rectal mass and retroperitoneal lymphadenopathy. Later, the patient underwent colonoscopy, and rectal mass was confirmed. Endoscopic ultrasound (EUS) was also positive for hypoechoic irregular mass extending into the muscularis propria with pelvic organ involvement. The lesion occupied 70% of the lumen. Histology analysis and immunohistochemistry (IHC) confirmed invasive, moderately differentiated SCC (Figure 1).

The tumor was staged as T3, N1, with involvement of circumferential free margin (CRM) or stage 3 B as per tumor-node-metastasis (TNM) classification. The patient was treated with concurrent chemoradiation with mitomycin/5-fluorouracil U and 54 Gy of radiation for two months. Later, a CT scan of the abdomen and pelvis revealed perforation at the tumor site posteriorly with involvement of the presacral space, and possibly right pelvic sidewall involvement. She then underwent a robotic proctectomy, end colostomy with en bloc radical total abdominal hysterectomy, and bilateral salpingo hysterectomy, with partial vaginectomy. Pathology was reviewed and showed no residual tumor consistent with complete response. The patient is currently in remission for two years.

## Discussion

The causative factors of rectal SCC remain unclear and many theories have been proposed. Chronic inflammation in ulcerative colitis, smoking, human immunodeficiency virus (HIV), enteric infections such as amebiasis or schistosomiasis has been considered as an association with rectal SCC in the past. The human papillomavirus (HPV) is the main culprit in causing dysplastic changes in any squamous epithelium. An association between HPV and SCC at various sites viz anus, head/neck, and cervix have been seen. Surprisingly, an association of HPV in the rectal SCC subtype has not been established. Nahas et al. studied 20 rectal cancer cases of SCC origin and did not show any evidence of HPV 6, 11, 16, or 18 [3]. The clinical symptoms in patients with rectal SCC are similar to those with the adenocarcinoma subtype. The most commonly reported symptom in any rectal cancer is rectal bleeding, altered bowel habit, abdomen pain, and weight loss. The time duration of symptoms can be variable, but many patients report a symptom history of weeks to months [4,5].

Colonoscopy or proctoscopy with biopsy is the key modality for the definitive diagnosis of rectal SCC. Sometimes, there can be difficulty in differentiating rectal SCC from the anus or small cell, poorly differentiated tumors on biopsy inferences [6]. Immunohistochemistry (IHC) is useful in characterizing such lesions. The most useful cytokeratins are CAM 5.2, AE1/AE3, and 34B12. CAM 5.2 differentiates rectal from anal lesions [5]. It specifically stains rectal SCC and adenocarcinoma and not anal squamous cell lesions [6]. Trans-rectal endoscopic ultrasound (EUS) is an important diagnostic approach for rectal cancer staging of all types. Correct staging helps to determine appropriate surgical treatment viz local excision or radical resection and the need for adjuvant therapy [6].

The proper management and guidelines of rectal SCC are still to be standardized due to its rarity. Rectal SCC has primarily been treated in the same manner as anal SCC because upfront chemo-radiotherapy has resulted in better overall survival (OS) in patients when compared to surgery followed by adjuvant chemotherapy or radiotherapy. Kommalapati et al. provided substantial data on treatment for rectal SCC cases from 1933 to 2016, illustrating OS of 86% with a chemo-radiotherapy group versus 48% for the surgery group [7]. Audeau et al. retrospectively studied 3405 cases of SCC of the rectum between 2004 and 2015 from the National Cancer Database (NCDB) [8]. It was noticed in the study that outcomes of rectal SCC were dependent mainly on age, sex, and therapy received. Patients between stages I and III who received chemoradiation therapy alone had 108 months of OS, and patients who received surgery alone had 76 months of OS [8]. Also, no statistical difference in OS was noted in groups that received surgery with adjuvant chemoradiation therapy [8].

Similar to rectal cancer, anal SCC was also managed by surgery [9]. However, the treatment of anal SCC was revolutionized with the Nigro protocol in 1974 by incorporating 3000 centigray (cGy) radiotherapy (RT) over three weeks, combined with 1000 mg/m^2^/day 5-FU chemo regimen as a continuous infusion over days one through four and repeated on days 29 through 32, with 10 mg/m^2^ mitomycin (MMC) delivered on days one and 29 [10]. Multiple studies have since demonstrated the benefit of the combined chemoradiotherapy (CRT) approach using 5-FU/MMC, as compared to RT alone of other chemotherapy combinations [11-14]. This combined-modality approach results in long-term local control, colostomy-free and overall survival (OS) in many anal cancer patients. Observing this shift in anal SCC treatment protocols, several reports have also increasingly focused on combining chemoradiotherapy (CRT) as the primary therapeutic approach for rectal SCC as well. Thus, reserving surgery for salvage. Lately, CRT as the primary intervention has consistently shown encouraging outcomes. Like anal SCC management guidelines, many reports of rectal SCC have also used a 5-FU-based CRT regimen, with a recommending radiotherapy dose between 5400 and 6000 cGy [15].

## Conclusions

Treatment approach from many trials has shown great promise for chemoradiation therapy as the mainstay treatment in rectal SCC. More study is required in the pathophysiology of this disease subtype and establish proper management guidelines. The purpose of this case report is to highlight and focus on the treatment response of this rare pathology.
